# Compliance with the Zero Suicide Initiative by Mental Health Clinicians at a Regional Mental Health Service: Development and Testing of a Clinical Audit Tool

**DOI:** 10.3390/nursrep13010003

**Published:** 2022-12-28

**Authors:** Joanne E. Porter, Elissa Dabkowski, Owen Connolly, Valerie Prokopiv

**Affiliations:** 1 Collaborative Evaluation & Research Group, Federation University Australia, Churchill 3842, Australia; 2 Mental Health Services, Latrobe Regional Hospital, Traralgon 3844, Australia

**Keywords:** zero suicide, suicide prevention, clinical audit, mental health, quantitative

## Abstract

Aim: The aim of this study is to investigate the compliance of mental health clinicians in applying the Zero Suicide (ZS) approach to their clinical practice in a rural and regional health community setting. Methods: A retrospective clinical audit of six mental health teams was undertaken at a single site. A clinical audit tool was developed and validated using a six-step approach. The data was extracted and analysed via descriptive and inferential statistics and compared to a specialised mental health team, experienced with the ZS approach. Results: A total of 334 clinical records were extracted for January, April, August, November 2019 and June 2020. The clinical audit and analysis confirmed that the mental health teams are not consistently using the assessments from their training and are therefore not implementing all of these elements into their practice. This could have implications for the risk formulation and treatment for people at risk of suicide. Conclusions: The use of a validated clinical audit tool can be beneficial to establish compliance with the mental health clinicians and to determine any areas requiring further improvement. Further education and reinforcement may be required to ensure consistency with incorporating the elements of ZS into everyday clinical practice.

## 1. Introduction

The World Health Organisation (WHO) estimates that a person dies by suicide every 40 s in the world, resulting in approximately 700,000 deaths a year [1]. Suicide is a global public health issue, affecting people across all life spans with its devastating consequences. Considerable efforts have been made to raise awareness of this public, but preventable health issue and to strengthen suicide prevention strategies [1]. Mental health clinicians play a key role in identifying, assessing, and implementing strategies to assist people at risk of completing suicide, with evidence revealing that 75% of individuals who died by suicide had visited a healthcare professional within three months preceding their death [2].

Suicide prevention requires a systems-approach, involving the implementation of multiple strategies and collaborating across sectors within a community [3]. As with any treatment approach, comprehensive evaluation is necessary to enable quality control and improve pre-existing strategies. Three key evaluation recommendations are: continuous monitoring of the implementation process of suicide prevention plans, evaluation of suicide prevention plans to form lessons learned for future efforts and evaluation of surveillance systems [4].

### Background

The Zero Suicide (ZS) initiative has potential to improve outcomes in people at risk of suicide, due to its structured holistic framework and organisational commitment [5]. The ZS model has seven elements for organisations to adopt, with the focus on improving patient safety outcomes, continuous quality improvement and the safety and support of clinical staff [5]. The ZS model was initially developed in 2011 by the Clinical Care and Intervention Task Force and the National Action Alliance for Suicide Prevention in the United States of America and has been implemented in over 200 healthcare organisations worldwide [6]. The elements of the ZS approach focus on system-wide culture change, workforce training, identifying individuals at risk of suicide, engaging and treating people at risk using evidence-based treatment, care transition, and improving policies and procedures through continuous quality improvement [5]. Studies have shown promising results with the implementation of the ZS approach. An analysis undertaken within a large Australian public mental health service reported a significant reduction in repeated suicide attempts (65%), as well as a longer time between attempts [7]. Similarly, the Centerstone in Tennessee reported a 65% reduction in the rates of suicide among patients after the implementation of the ZS framework [6]. Despite these promising statistics, further research is recommended to establish the efficacy of the implementation of the ZS framework, especially in an Australian setting [7,8,9].

The introduction of the ZS approach to a community mental health service at a large regional mental health unit in Victoria, Australia provided an opportunity to assess the efficacy of its implementation. In an Australian context, specialised mental health care can be accessed through emergency departments, residential mental health and community mental health services, which is funded by state and territory governments [10]. A Suicide Prevention Pathway (SPP) was introduced to a single Community Mental Health team located two hours from the main regional mental health unit, in which each step of the ZS framework was embedded within the hospital’s current processes. This community mental health team consist of a range of health professional specialising in mental health including mental health nurses, occupational therapist, social worker, clinical psychologist, with overarching support by the treating psychiatrist. All staff attended two days of SPP training in February 2019 with periodical refresher in-services provided to staff thereafter. The purpose of the SPP was to improve suicide prevention practices through improving the consistency and quality of assessments, formulation safety planning and consumer education. The authors developed a ZS clinical audit tool to appraise patient clinical records to assess the compliance of mental health clinicians in adopting these practices. A clinical audit is defined as “*a quality improvement process that seeks to improve patient care and outcomes through systematic review of care against explicit criteria and the implementation of change*” [11]. Hospital records can provide a valuable and rich data source of information to compile data and to enhance our knowledge on suicide [4]. To the authors’ knowledge, no other study has specifically investigated the compliance of mental health clinicians in the adoption of the ZS model within their clinical practices.

The aim of this study is to investigate the effectiveness of the ZS model through conducting a clinical audit to establish the compliance of the mental health clinicians at a regional mental health service. The authors intend to identify any potential areas of improvement that may assist in the delivery of these practices to people at risk of suicide. Given the significance of this public health issue, considerable efforts are necessary to contribute to the current evidence base regarding suicide prevention programs and to address any gaps in the literature.

## 2. Methods

### 2.1. Design

This study was a single-site retrospective clinical audit, using a quantitative descriptive design. A clinical audit tool was developed and used to extract data from client records to assess the implementation of the Zero Suicide prevention approach based on the Collaborative Assessment of Suicide Events (CASE)—Shawn Shea Model, in a single large regional hospital.

### 2.2. Audit Tool Development

The clinical audit tool (Appendix A) was developed using the six-step process as outlined by McConnell-Henry et al. [12]. After the audit aims were identified (step one), the 41-item data extraction tool was developed by the research team in consultation with the auditors (step two). Step three involved establishing content validity index (CVI), in which the data extraction tool was sent to five experts with extensive experience. These experts included a Professor of Mental Health, who was both a content and design expert, an Executive Director of a regional mental health service, a mental health practitioner together with two academic design experts. The 41-item audit tool was tested for clarity and relevance, in which each item was ranked highly relevant by all five content and design experts. When assessing the CVI of a focused data set, an overall scale is considered to be valid with an index of ≥0.90 [12]. In this study, the Scale Content Validity Index Average (S-SCI/Ave) for all items was 0.92, therefore no changes were required of the 41 items. Using expert feedback, minor amendments were made to the tool such as the inclusion of the partial column and the low, medium and high ratings scale even though this is not considered to be part of the ZS approach. Step four and step five involved pilot testing and training using the audit tool, in which the two auditors each completed two individual clinical records and compared the findings. Interrater reliability was established for this tool, in which the two auditors underwent clinical audit training by author JP to ensure uniformity in the audit. The two auditors also have extensive experience in the mental health field and were the sole auditors. Step six involved establishing the sample size.

### 2.3. Data Collection

Patient medical records were accessed by members of the project team affiliated with the mental health service for five different time periods following ethical approval. The data was extracted from medical files from the regional Community Mental Health Services. The sample size of the population was calculated to be *n* = 292 based on a predicted population of 1202 case records with 95% confidence intervals. A cluster sampling strategy was involved, in which the research team opted to extract data from the months of January 2019 (pre SPP staff training), April 2019, August 2019, November 2019 and June 2020. These months were specifically selected by the research team to evaluate the long-term compliance of mental health clinicians in implementing the SPP. Data from the Hospital Outreach Postsuicidal Engagement (HOPE) program was used for comparison. The role of the HOPE clinicians is to provide support for people after discharge from emergency departments (ED) who are identified as at-risk of suicide or for those who express suicidal ideation and/or repeated intentional self-harm. The HOPE team had previously embedded the ZS approach to their practice and are considered to be the practice benchmark for the ZS approach within this health service. After data extraction, each audit tool was deidentified and coded, before being electronically scanned and sent to the research team for analysis. Ethical approval was granted from the hospital ethics committee and the University human ethics committee prior to extraction of data using the clinical audit chart tool (Project No. 2020-20 HREA and A20-070).

### 2.4. Data Analysis

The data was collated and entered into a SPSS software (IBM SPSS Statistics for Windows, Version 26.0, IBM Corp, Armonk, NY, USA) file, cleaned and checked for missing data. Basic demographic data such as age, gender, referral origin, mental health team and nature of presentation was analysed using descriptive statistics. After earlier consultation with an expert statistician, it was decided to use descriptive and inferential analysis through the computer program IBM SPSS V25. Pearson-Chi squared statistics were incorporated to establish statistical significance between the expected and observed frequencies of the data. Initial comparisons were made between the extraction points, followed by a combining of the data for analysis.

## 3. Results

### 3.1. Demographics

A total of 336 charts were extracted from the selected time periods, in which 334 records constituted the final data set. The remaining two charts were excluded due to incomplete or missing data. Table 1 details the demographical data, in which the mean age of the clients was 39.8 years, with the majority of clients aged between 25–45 years of age, ranging from 8–97 years. The most frequent group was the 25 to 35-year age group (*n* = 73) followed by the 36 to 45-year age group (*n* = 60). Gender related data revealed that 52% (*n* = 171) of clients identified as male, 47% (*n* = 157) of clients identified as female and 1% (*n* = 4) of clients identified as intersex, and two charts had missing data.

The clinical audit identified six main mental health teams at the regional hospital: Acute Community Intervention Service (ACIS), Aged Persons Mental Health Service (APMHS), Child and Youth Mental Health (CYMHS), Recovery, Prevention/Recovery and HOPE. The ACIS team received the most referrals, followed by the APMHS and CYMH teams. The greatest number of referrals occurred during June 2020. The HOPE data is presented as a combined data set with most of the referrals occurred in June 2020. It is possible that natural disasters such as the bushfires and COVID-19 may have impacted the regional community, contributing to the increase of referrals to mental health services.

The ED was the most common point of referral of clients with 55% (*n* = 182) followed by ACIS (17%, *n* = 56) and a general practitioner (14%, *n* = 47). Data from the treatment plans showed that 53% (*n* = 177) of cases were referred to intake, indicating that clients were referred for further mental health services. A total of 25% (*n* = 85) of clients were referred for crisis assessment.

Specific data was collected for each age category, including official diagnoses, identified suicide drivers and number of cases involving suicide attempts, suicidal ideation, non-suicidal self-injury and self-harm. Four clients under the age of ten years were identified in the data, with depression recognised as the key diagnosis in 50% of cases, 75% of cases did not specify suicide drivers. In the category 11–17 years of age (*n* = 34), suicidal ideation occurred in 50% of cases, with 23.5% of clients specifying non-suicidal self-injuries. The 34 clients aged 11–17 years, had no formal diagnoses noted for 44% of cases, with 27% of clients diagnosed with depression and 27% with behavioural disorders. For 53% of the clients in this age group, no suicidal drivers were specified with 20% identifying family and friendship stressors as their primary suicide driver.

In the age group 18–24 years (*n* = 46), 41% of cases had no formal diagnosis, with 22% having a diagnosis of depression, followed by 18% of cases with personality disorders. Suicidal ideation was reported in 74% of cases followed by active suicide attempts in 28% of cases. In this age group, 15% of clients reported self-harm and 13% indicated non-suicidal self-injuries. A total of 37% of clients did not identify specific suicide drivers with a further 19% of clients identifying family and friendship stressors as specific suicide drivers. In the age group 25–35 years (*n* = 73), 28% of cases had no formal diagnosis, 18% had a diagnosis of depression, and 14% had a diagnosis of adjustment disorder. Suicidal ideation was expressed in 52% of cases and 16% of cases reported a suicide attempt. A total 49% of cases reported no specific suicide drivers, followed by 29% of clients in this age group reporting family and friendship breakdown as the main driver.

Depression was the most predominant diagnosis for the 36–45-year age group (*n* = 60) at 26% of cases, followed by schizophrenia in 21% of clients. A total of 50% of cases in this age group indicated suicidal ideation, with 13% reporting suicide attempts and 10% of cases reporting non-suicidal self-injuries. A total of 45% of clients were identified as having no specified suicide drivers and 30% of cases reported their main suicide driver was due to family/friendship or relationship breakdown. Personality disorders was the predominant diagnosis in the age group of 46–55 years (*n* = 43) with 28% of cases. This was followed by depression in 25% of clients and schizophrenia at 21%. In 54% of cases, no suicide drivers were specified. Suicidal ideation occurred in 51% of cases in this age category followed by 14% reporting a suicide attempt. In the age group 56–65 years (*n* = 27), 38% of clients did not have a formal diagnosis. For 44% of cases, no suicide drivers were specified followed by family/friendship stressors, financial stressors and medical factors including auditory hallucinations, self-harm, each at 15% correspondingly. Suicidal ideation was high in this age group in 52% of people, followed by 22% of people reporting suicide attempts. Non-suicidal self-injuries was identified in 11% of cases followed by self-harm at 7%.

Depression was the significant diagnosis for people in the age category of 66–75 years (*n* = 22) with a reported 33% of clients. Suicide drivers were not specified in 72% of cases and 27% of people in this age category expressed suicidal ideation. For the 76–85-year-old age group (*n* = 21), 33% had a diagnosis of dementia followed by 28% with no formal diagnosis. In 85% of cases nil suicide drivers were identified. A total of 19% in this age group reported suicidal ideation with 14% reporting they had had a suicide attempt. In the >86-year age group (*n* = 4), 50% of clients had expressed suicidal ideation. A total of 75% of cases had no formal diagnosis and 25% had a diagnosis of dementia.

### 3.2. Audit Chart Findings

The audit tool was divided into six distinct sections which included screening, assessment, risk formulation, safety plan, prevent access to lethal means and client and carer education. These sections were analysed using the HOPE data (*n* = 35) as the practice benchmark in which the performance of the combined mental health teams (*n* = 299) were compared to that of HOPE’s screening.

### 3.3. Screening and Assessment

The data pertaining to mental health practitioners screening clients for suicidality indicates that the HOPE team’s performance exceeded expectations, 25 compared to the expected value of 7.4. This is in direct contrast to the other mental health teams who were expected to score at least 63.6 in this variable but only achieved a score of 46 (χ^2^ 58.793, *p*-value 0.001). The assessment section reflected the key components of the Collaborative Assessment of Suicide Events (CASE), based on the Shawn Shea Model taught in the SPP education (Table 2). The HOPE team performed better than expected for all these variables, demonstrating that they are conducting thorough assessments for people who are at risk of suicide.

The statistic worth noting is that of the observed 154 people that were identified as requiring an assessment at screening, only 97 people received a completed assessment, which indicates that an assessment was not completed for 57 people who were deemed to be at risk of suicide. The other mental health teams partially explored some of these variables, such as the context and details of suicide behaviour (method), plans and degree of preparation, recent, past and previous events, enquiring about other suicide methods and identifying drivers of suicidality. Table 3 demonstrates the differences at each extraction point between assessment indicated and assessment completed.

The results from Table 3 demonstrate that the likelihood of a person at risk of suicide receiving a completed assessment, depended on the specific mental health team. For example, the HOPE team, who specialise in caring for high risk clients, completed a total of 35 assessments for which 36 assessments were indicated. In comparison, there were mixed results amongst other mental health teams, in which the completion rate ranged from 46% to 77%. As an example, in August 2019 there were a total of 66 client records extracted across combined mental health teams in which 35 were identified as requiring a ZERO assessment. The clinical audit revealed that only 16 clients received an assessment in August 2020, at a compliance rate of 46%. The overall compliance rate of the mental health teams (excluding the HOPE team) was 63%.

A Pearson Chi-Squared test of independence was performed to examine the relationship between the assessment indicated, the assessment completed and the extraction points. This statistical test is appropriate for comparing three factors of nominal data. The relationship between the extraction points and assessment indicated was significant, χ^2^ (5, *N* = 335) = 39.104, *p* = 0.000. A statistically significant relationship was also established between the assessment completed and the extraction points, χ^2^ (5, *N* = 335) = 71.093, *p* = 0.000. However, there was no statistical significance between the two variables assessment indicated and assessment completed throughout the duration of the data collection period, χ^2^ (5, *N* = 335) = 6.391, *p* = 0.270. Staff training commenced in February 2019, yet it does not appear to have influenced the rate of completed assessments. In comparison, the HOPE team maintained 100% compliance between indicated and completed assessments, throughout the data collection period. Further research is required to explore the reasons for the decreased compliance with completed assessments by the other mental health teams.

### 3.4. Risk Formulation

The HOPE team demonstrated excellent compliance when assessing the risk state and risk status, as well as evaluating foreseeable changes, available resources and internal/external drivers. As shown in Table 4, the observed frequencies (*n* = 32, 30, 31, 21 and 19) far exceeded the expected frequencies (*n* = 4.4, 4.6, 3.9, 2.7 and 2.9) in the subsequent columns, with the *p*-value less than 0.001, indicating statistical association. Similar to the other sections, the other mental health teams did not complete the risk formulations as expected; however, there was partial compliance with the risk formulations for three of the variables, risk state, risk status and foreseeable changes (*n* = 23, 25 and 11), as compared to the expected partial frequencies (*n* = 22.4, 25.1 and 10.7).

There was not a statistically significant association between the ratings of low, medium or high used with HOPE and other mental health teams (*p* = 0.30). This indicates that there is not a relationship between using this variable and belonging to either HOPE or the other mental health teams. Of concern is that the practice of using this variable as part of a suicide prevention assessment is outdated. The fact that HOPE and other mental health teams are still incorporating these ratings into their assessments, indicates that further education is needed to ensure that all mental health clinicians are using current evidence-based practice.

### 3.5. Safety Plan

The HOPE team demonstrated high compliance when it came to implementing the correct safety plan, as well as involving the client and family. Table 5 shows that there was a statistical association between these variables, along with the safety plan identifying suicide drivers with the *p*-value less than 0.001. Most importantly, the accuracy of these safety plans was noted with the observed frequency (*n* = 33) far exceeding the expected frequency (*n* = 4.6).

The observed frequencies for these variables (*n* = 11, 5 and 0) were significantly less than the expected frequencies (*n* = 39.4, 18.8 and 22.4). This correlates with the demographic data which identified that specific suicide drivers were not thoroughly investigated. The HOPE team was highly compliant with completing safety plans with the client and family, as well as identifying drivers.

### 3.6. Preventing Access to Lethal Means

The HOPE team demonstrated exceptional performance in the two variables under preventing access to lethal means. The observed frequencies (*n* = 19) were greater than the expected frequencies (*n* = 2.1) in identifying lethal means for the HOPE team and the subsequent safety plans for clients including the lethal means interventions were deemed to be appropriate. The other mental health teams demonstrated significant shortcomings with preventing access to lethal means.

### 3.7. Client and Carer Education

There was a statistically significant association between adequate client and carer education provided by the HOPE team, as compared to the other mental health teams. The observed frequencies (*n* = 5 and *n* = 1) were greater than the expected frequencies (*n* = 1.3 and *n* = 0.1) for the HOPE team in the two variables regarding the safety plan discussions with both client and family, as well as the provision of the Beyond Blue information books. The observed frequencies (*n* = 7 and *n* = 0) for the other mental health teams were less than the expected frequencies (*n* = 10.7 and *n* = 0.9) as demonstrated in Table 5.

## 4. Discussion

The objective of this study was to conduct a clinical audit to determine the compliance of mental health clinicians in implementing the ZS approach at a regional mental health service. The use of a validated clinical audit tool was an essential component of the clinical audit to determine the areas of the SPP steps that specifically required further improvement. This audit was necessary as it provided an opportunity to identify areas that mental health clinicians would benefit from further education and training support. This aligns with evidence that recommends organisations create processes to assess compliance to the SPP and to evaluate outcomes on a systems, policy, and individual basis [5]. This commitment to continuous quality improvement is characteristic of the final element within the ZS framework [5].

The standout statistic in this clinical audit was the lack of specified suicide driver identified for each of the age groups. This indicates that the specific suicide drivers were not thoroughly investigated by the mental health clinician at each assessment, potentially leading to gaps in risk formulation and treatment. A comprehensive suicide risk formulation should be completed when a person screens positive for suicidal risk, with all staff utilising the same risk formulation model [5]. Another possibility was that the specific suicide drivers were not documented accurately, which again indicates a potential deficit in following the SPP pathway. Interestingly, many of the clients from the clinical audits did not have a formal mental health diagnosis. This was evident in the 18–24 year age group, in which 41% of cases had no formal diagnosis. These clients may have accessed mental health services for the first time or may have previously accessed services without complying with the follow-up recommendations. The high number of cases without a formal diagnosis is concerning, especially since some diagnoses such as schizophrenia could be under-represented in our findings. Suicide is the largest contributor to decreased life expectancy in those with schizophrenia and it is imperative that clinicians identify risk factors in this cohort and offer comprehensive assessments [13,14]. This is also important for those newly diagnosed with dementia, as there is an elevated suicide risk during the first twelve months after diagnosis, with the highest risk in patients aged 65 to 74 years [15]. The findings of Stapelberg et al. [7] emphasise the importance of identifying vulnerable individuals who may have presented for the first time and to offer suitable services. This may provide greater improvements for the first-time presenter and may help to avoid multiple presentations in the future [7].

The HOPE team were 100% compliant in completing assessments for all clients who were identified as requiring an assessment at screening. These findings were viewed favourably and indicates that the HOPE team is conducting thorough assessments and exceeding the expected frequencies. This is anticipated as the HOPE team are known as the acute crisis management team within this health service. As evident from the audit, all six mental health teams within this health service receive referrals for at-risk patients and are expected to conduct comprehensive assessments. The difference is the HOPE team receives these types of referrals more frequently than the other mental health teams. The qualitative analysis of this project identified that mental health clinicians from a variety of teams required regular, ongoing suicide prevention education that was tailored to their area of practice [16]. Even still, areas of improvement identified for the HOPE team include further exploration of the behavioural incident (events over the prior 48 h) and further exploring context and details of suicidal behaviour including location. In keeping with the ZS framework, further education and training are recommended to improve full compliance by the other mental health teams.

Although this clinical audit demonstrated the areas of improvement needed within mental health teams at a regional mental health service, this should not be viewed as a criticism of the mental health clinicians. The purpose of this clinical audit is not to vilify the community mental health clinicians, but to provide recommendations for improvement and areas requiring further education. This aligns with current literature which recommends implementing a Restorative Just Culture alongside the ZS framework in a hospital or health service [17]. The just culture is imperative to gaining the clinicians’ trust and commitment to organisational changes [17]. Although there is need for further research into the feasibility and effectiveness of large-scale implementation of the ZS approach [9], this study contributes to the literature from a large single health service in a regional and rural setting.

### 4.1. Implications for Practice

The findings of this study reinforce the importance of regular evaluation and implementing continuous quality improvement processes to ensure that mental health clinicians are utilising the latest evidence-based research when working with people at risk of suicide. This detailed clinical audit form has the potential to be used by other mental health services that use the ZS approach, to assess clinician compliance. Recommendations for future research include longitudinal studies to investigate the long-term compliance of the ZS approach. Another possible avenue is to investigate the mental health clinicians’ perspective of the ZS approach using a qualitative lens.

### 4.2. Limitations

A limitation to the findings of this study is the consistency and experience of mental health clinicians using the ZS approach. The HOPE team are a specialised unit, who regularly engage with people at risk of suicide. In comparison, the other mental health teams may not have regular contact with people at risk of suicide, therefore this inconsistency may have influenced the accuracy of the findings. Another potential limitation is the data collection of this study occurred at specific time points at several sites within a regional mental health service. It is possible that the findings of this study may not be generalised to metropolitan or other regional settings

## 5. Conclusions

In all six sections of the clinical audit, which was based on the CASE—Shawn Shea Model, the HOPE team demonstrated a statistically significant association between these variables and their ability to implement these elements into their practice. The clinical audit and subsequent descriptive analysis confirmed that the other mental health teams are not consistently using the assessments from the SPP training and are therefore not implementing all of these elements into their practice. Further education and reinforcement of the Zero Suicide approach may be needed to facilitate consistency between mental health clinicians in assessing and managing people at risk of suicide. The development of the clinical audit tool proved to be an effective means to evaluate the compliance of mental health clinicians.

## Figures and Tables

**Table 1:** Chart audit demographics.

	Chart Audit
Age (%)	mean 39.8 (±19.943)
<10 years	4 (1.2%)
11–17 years	34 (10.2%)
18–24 years	46 (13.8%)
25–35 years	73 (21.9%)
36–45 years	60 (18.0%)
46–55 years	43 (12.9%)
56–65 years	27 (8.1%)
66–75 years	22 (6.6%)
76–85 years	21 (6.3%)
>86 years	4 (1.2%)
unknown age	2 (0.6%)
Gender (%)	
Female	157 (47%)
Male	171 (51.2%)
Intersex	4 (1.2%)
Mental Health Team (%)	
CYMHS	43 (12.9%)
Prevention/Recovery	1 (0.3%)
ACIS	205 (61.4%)
APMHS	44 (13.2%)
Recovery	2 (0.6%)
HOPE	35 (10.5%)
Nil	4 (1.2%)

**Table 2:** Assessment. Key—Green: above expected and Orange: below expected.

Assessment	HOPE						Mental Health Teams						Pearson Chi Sq	*p*-Value
	Observed			Expected			Observed			Expected				
	Yes	Part	No	Yes	Part	No	Yes	Part	No	Yes	Part	No		
Is an Assessment indicated at screening?	35	n/a	0	19.8	n/a	15.2	154	n/a	145	169	n/a	130	29.995	0.001
Was an assessment completed?	35	n/a	0	13.8	n/a	21.2	97	n/a	202	118	n/a	181	59.830	0.001
Behavioural incident (events over prior 48 h)	3	5	27	1.2	2.7	31.1	8	21	270	9.8	23.3	265.9	6.040	0.049
Withheld and reflected intent are present	16	9	10	3.7	5.1	26.2	19	40	240	31.3	43.9	223.8	60.758	0.001
Context and details of suicide behaviour—Location	12	2	21	2.1	0.9	32	8	7	284	17.9	8.1	273	57.805	0.001
Context and details of suicide behaviour—Method	24	0	11	9.4	2.3	23.3	66	22	211	80.6	19.7	198.7	34.937	0.001
Identifies any of frequency, intensity, duration of thoughts	10	16	9	3.1	4.9	26.9	20	31	248	26.9	42.1	230.1	57.858	0.001
Plans and degree of preparation	22	1	12	4.9	2.2	27.9	25	20	254	42.1	18.8	238.1	76.956	0.001
Recent, past and previous events are explored	29	6	0	6.5	5.9	22.6	33	50	216	55.5	50.1	193.4	112.352	0.001
Were other suicide methods enquired about	14	1	20	2.5	1.2	31.3	10	10	279	21.5	9.8	267.7	63.189	0.001
Drivers of suicidality identified	33	2	0	9.3	5.2	20.4	56	48	195	79.7	44.8	174.6	92.19	0.001
Collateral information	2	2	31	1	0.8	33.1	8	6	285	10	8	316	2.915	0.233

**Table 3:** Comparing extraction points of assessments indicated and assessments completed.

Extraction Point—All Mental Health Teams	Total Clients	Assessment Indicated from the Total	Assessment Completed Following Indication
January 2019	63	*N* = 36, 57%	*N* = 28, 77%
April 2019	42	*N* = 19, 45%	*N* = 11, 58%
August 2019	66	*N* = 35, 53%	*N* = 16, 46%
November 2019	46	*N* = 31, 67%	*N* = 22, 71%
June 2020	82	*N* = 32, 39%	*N* = 19, 59%
HOPE team	36	*N* = 35, 97%	*N* = 35, 100%
Total number of audits	335	*N* = 153, 46%	*N* = 96, 63%

**Table 4:** Risk Formulation. Assessment. Key—Green: above expected and Orange: below expected.

Risk Formulation	HOPE						Mental Health Teams						Pearson Chi Sq	*p*-Value
	Observed			Expected			Observed			Expected				
	Yes	Part	No	Yes	Part	No	Yes	Part	No	Yes	Part	No		
Risk State	32	2	1	4.4	2.6	28	10	23	266	37.6	22.4	239	222.547	0.001
Risk Status	30	3	2	4.6	2.9	27.5	14	25	260	39.4	25.1	234.5	182.536	0.001
Foreseeable Changes	31	1	3	3.9	1.3	29.9	6	11	282	33.1	10.7	255.1	238.998	0.001
Available Resources	21	8	6	2.7	2.8	29.4	5	19	275	23.3	24.2	251.6	168.345	0.001
Internal & External Drivers	19	9	7	2.9	2.9	29.1	9	19	271	25.1	25.1	248.9	131.056	0.001
Are ratings of Low, Medium or High used?	4	n/a	31	2.5	n/a	32.5	20	n/a	279	21.5	n/a	277.5	1.055	0.300

**Table 5:** Safety planning, prevention of access to lethal means and client and carer education. Assessment. Key—Green: above expected and Orange: below expected.

Safety Plan	HOPE						Mental Health Teams						Pearson Chi Sq	*p*-Value
	Observed			Expected			Observed			Expected				
	Yes	Part	No	Yes	Part	No	Yes	Part	No	Yes	Part	No		
Was the Correct Safety Plan Completed?	33	n/a	2	4.6	n/a	30.4	11	n/a	288	39.4	n/a	259.6	224.883	0.001
Was it completed with the Client & Family?	16	1	18	2.2	0.2	32.6	5	1	293	18.8	1.8	278.4	107.288	0.001
Did the Safety Plan identify drivers?	25	4	6	2.6	0.6	31.8	0	2	297	22.4	5.4	271.2	257.094	0.001
**Prevent Access to Lethal Means**														
Were the identified Lethal Means covered in the safety plan?	19	1	15	2.1	0.4	32.5	1	3	295	17.9	3.6	277.5	163.716	0.001
Were the Lethal Means Interventions appropriate?	13	6	16	1.8	0.7	32.5	4	1	294	15.2	6.3	277.5	130.501	0.001
**Client and Carer Education**														
Was the safety plan discussed with both client and family?	5	n/a	30	1.3	n/a	33.7	7	n/a	292	10.7	n/a	288.3	12.906	0.001
Were the Beyond Blue information books provided and discussed?	1	n/a	34	0.1	n/a	34.9	0	n/a	299	0.9	n/a	298.1	8.569	0.001

## Data Availability

The data presented in this study are available on reasonable request from the corresponding author. The data are not publicly available due to privacy reasons.

## Appendix A. ZERO Suicide Prevention Pathway (SPP) Audit Chart

**Audit Tool Number**		**UR/Case Identifier**				
**Age**		**Gender**				
**Screened date**		**Assessment date**				
**Suicide drivers**		**Diagnosis**				
**Referral origin**		**MH team** Mental health/police response □ AGED □ Recovery □ Child and Youth Mental Health □ Acute Community Intervention Service (ACIS) □				
Assessment outcome No further action □ Refer to GP □ Refer to ACIS □ Refer to AGED □ Refer to Child and Youth Mental Health □ Refer to Recovery □ Other □ _____________________________						
**Suicide attempt. Yes/No**		**Suicide ideation. Yes/No**				
**Self-harm. Yes/No**		**Non-Suicidal Self-Injury (NSSI). Yes/No**				
**SPP steps**			Yes	Partial *	No	Comments
**Screening**	Is there an indication of screening questions asked?					
	
**Assessment**	Is an assessment indicated at screening?					
	Has an assessment been completed?					
	Are the key components of CASE * approach present?					
	• Behavioural incident (events over prior 48 h)					
	• Withheld and reflected intent are present					
	Does the assessment of suicide or self-harm include?					
	• Context and details of suicide behaviour—Location					
	• Context and details of suicide behaviour—Method					
	• Identifies any of frequency, intensity, duration of thoughts					
	• Plans and degree of preparation,					
	• Recent, past and previous events are explored.					
	• Were other suicide methods enquired about e.g., hanging, firearms					
	• Drivers of suicidality identified					
	• Collateral information.					
	
**Formulation**	Are the elements of risk formulation included?		Yes	Partial	No	
	• Risk state,					
	• Risk status,					
	• Foreseeable changes,					
	• Available resources					
	• Internal and external drivers,					
	• Are the ratings of low, medium or high used?					
	
**Safety planning**	• Was the correct suicide safety plan completed					
	• Was it completed with the client and family involved					
	• Did the safety plan detail adequately identify drivers?					
	
**Lethal Means Interventions**	• Were the lethal means identified in assessment covered in the safety plan					
	• Are the lethal means intervention appropriate					
	
**Client and family education**	• Was the safety plan discussed with both client and family					
	• Were the Beyond Blue information books provided and discussed					
						
* CASE—Collaborative Assessment of Suicide Events- Shawn Shea Model taught in the SPP education; * Partial—if shaded, not applicable, do not tick.						
